# Ascorbic Acid as an Adjuvant to Unbleached Cotton Promotes Antimicrobial Activity in Spunlace Nonwovens

**DOI:** 10.3390/ijms23073598

**Published:** 2022-03-25

**Authors:** Judson Vincent Edwards, Nicolette T. Prevost, Dorne Yager, Robert Mackin, Michael Santiago, SeChin Chang, Brian Condon, Joseph Dacorta

**Affiliations:** 1 Southern Regional Research Center, Agricultural Research Service, United States Department of Agriculture, 1100 Robert E. Lee Blvd., New Orleans, LA 70124, USA; nicolette.prevost@usda.gov (N.T.P.); robert.mackin@usda.gov (R.M.); michael.santiago@usda.gov (M.S.); sechin.chang@usda.gov (S.C.); brian.condon@usda.gov (B.C.); 2 Department of Surgery, Virginia Commonwealth University School of Medicine, Richmond, VA 23298, USA; dorne.yager@vcuhealth.org; 3 H&H Medical, Williamsburg, VA 28135, USA; jda@gohandh.com

**Keywords:** antimicrobial activity, ascorbic acid, cotton

## Abstract

The development of affordable, effective, and environmentally friendly barrier fabrics is a current goal in antimicrobial textile development. The discovery of new routes to achieve non-toxic naturally occurring molecules with antimicrobial activity is of interest in the development of materials that promote wound healing, improve hygiene, and offer protection against nosocomial infection. Highly cleaned and sterile unbleached cotton has constituents that produce hydrogen peroxide at levels commensurate with those that favor cell signaling in wound healing. Here, we show the antimicrobial and antiviral properties of spunlaced griege cotton-containing nonwovens treated with ascorbic acid formulations. The mechanism of action occurs through the promotion of enhanced hydrogen peroxide activity. The levels of hydrogen peroxide activity afford antimicrobial activity against Gram-negative and Gram-positive bacteria and antiviral activity against MS2 bacteriophages. Spun-bond nonwoven unbleached cotton was treated with ascorbic acid using traditional pad-dry-cure methods. An assessment of antibacterial and antiviral activity against *Staphylococcus aureus*, *Klebsiella pneumoniae*, and MS2 bacteriophages with the AATCC 100 test method showed a 99.99% inhibitory activity. An approach to the covalent attachment of ascorbic to cellulose through citric acid crosslinking chemistry is also discussed. Thus, a simple, low-cost approach to antimicrobial and antiviral cotton-based nonwovens applicable to dressings, nosocomial barrier fabrics, and face masks can be adopted by combining ascorbic acid with spunlace greige cotton nonwoven fabrics.

## 1. Introduction

The current worldwide consumption of both synthetic and natural fibers by the textile industry continues to grow and encompass a wide range of potential uses for antimicrobial application [1]. However, commercial approaches used to impart antimicrobial activity to textiles have largely been based on the use of synthetic small molecules that are affixed by diffusion or grafting to a fiber [2]. Thus, the development of antimicrobial textiles using naturally occurring, non-toxic molecules is of increasing interest. Moreover, this is relevant to new applications for human use in health and hygiene, including wound dressings and medical textiles inclusive of body-contacting materials, where nosocomial antibiotic resistance is becoming an issue of concern [3].

In recent years, ascorbic acid has received increased attention due to its in vivo activity as a pro-oxidant directed to human diseases such as cancer [4]. The underlying mechanism of activity is thought to be based on hydrogen peroxide and mediated by the Fenton reaction. In addition, it is noteworthy that ascorbic acid, has been used as a food preservative. [5]. Moreover, ascorbic acid demonstrates some antibacterial activity singularly and in combination with other organic compounds and metals. However, prior studies on this topic report only partial inhibitory activity against bacterial growth. Thus, we outline approaches used to confer 99.99% antibacterial activity against Gram positive bacteria and negative activity through the formulation of ascorbic acid with greige cotton nonwovens.

It is also noteworthy that there is increasing interest in the development of anti-infective wound dressings, and this is especially the case for bleeding-control dressings, where the prevention of infection is a concern in prolonged field care. Recently, we reported on the design and preparation of bleeding-control dressings based on spunlaced blends of greige cotton [6]. Here, we describe a process used to confer antimicrobial activity on a bleeding-control dressing. In addition, this study has relevance for the generalized process of preparing ascorbic acid-treated greige cotton nonwovens designed to confer antibacterial as well as antiviral activity to a barrier fabric. To this end, we also explore routes to achieve the covalent attachment of ascorbate to cellulose and the resulting activities.

## 2. Results

### 2.1. Antimicrobial Activities of Treated Fabrics

The impetus for this study was to determine whether the commercial hemostatic fabric, TACGauze, could be modified by traditional finishing chemistry treatments to create a fabric that retains its hemostatic function but also possesses beneficial antibacterial and antiviral properties.

Table 1 defines TACGauze, the fabric identification, and its treatments/methods with ascorbic acid. The table also identifies 100% cotton woven and nonwoven fabrics and defines their treatments. Initially, TACGauze (TGz) was treated with ascorbic acid, resulting in an antibacterial prototype termed BIOGauze (BGz). Table 2 shows that BGz, a formulation of a greige cotton-based spunlaced nonwoven material with ascorbic acid, produces a 99.99% antibacterial activity.

Table 2 displays the antimicrobial results, assessed using the AATCC 100 test method, for most of these fabrics. Both pad-dry, pad-dry-cure, adsorption, and crosslinking methods of application of ascorbic acid formulations show a 99.99% inhibition of both Gram-negative and Gram-positive bacteria. The study contrasts pad-dry-cure lab and pilot labscale (L and PL) methods with spray-dried methods (S1&S2) for TACGauze (TGz). Pad-dry-cure, adsorption, and crosslinking (X1) applications of ascorbic acid formulations showed a 99.99% inhibition of both Gram-negative and Gram-positive bacteria. However, notably pad pressure has a slight influence on activity from 5 psi to 30 psi, representing a decrease of 0.35% against *K. Pneumonia*. On the other hand, the application of spray-drying resulted in a 99.9% inhibition of both Gram-negative and -positive bacteria. Thus, spray-dry application methods, though one order of magnitude less effective against both strains of bacteria, are still within the limits required to sanitize a surface. This is also important in light of its applicability to commercial nonwoven material processes. However, the application of spray-drying to plain-weave 100% cotton woven fabrics (GCS1 and S2) had significantly less of an effect—i.e., a 94.8 percent inhibition against S. aureus. Thus, the differences in activity are notable in light of the change in antibacterial activity that occurs when ascorbic acid is applied to a nonwoven greige cotton fabric versus a woven fabric.

The application process and ascorbic acid stoichiometry were found to confer activity at add-on amounts of less than one percent. Moreover, the antibacterial activity observed is consistent with previous levels of hydrogen peroxide observed within a range reported to be antibacterial [7,8]. This led to an investigation of the mechanism of action of multiple ascorbic acid-based dressing formularies that could potentially be used as barrier fabrics for antimicrobial and antiviral activity.

### 2.2. Hydrogen Peroxide Activity

The results of the antimicrobial activity of the fabrics led to an effort to delineate the mechanism of action of multiple ascorbic acid-based dressing formularies that could potentially be used as barrier fabrics for antimicrobial and antiviral activity.

However, the observation of levels of hydrogen peroxide generation associated with antibacterial activity prompted us to examine the supplementation of the fabric with ascorbic acid. Here, we demonstrate this under different finishing chemistry conditions with the goal of developing antimicrobial and antiviral fabrics.

**Figure 1:**
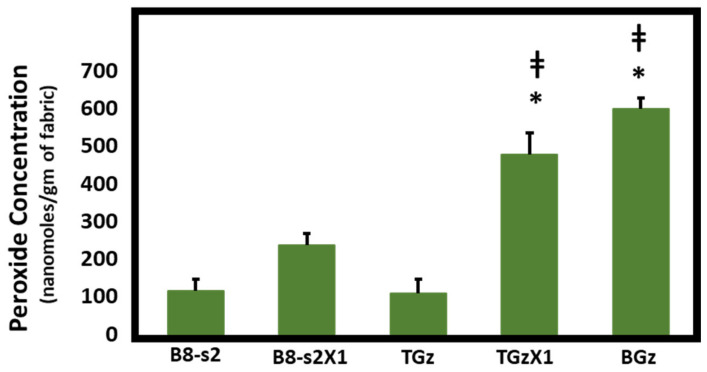
Comparison of hydrogen peroxide production. Fabrics (50 mg) were incubated overnight in 2 mL of H_2_O at room temperature. Aliquots were removed and H_2_O_2_ content was determined using the ferrous oxidation-xylenol orange assay in quadruplicate, *n* = 4. Error bars represent Standard Error of Mean (SEM). Results were analyzed by ANOVA. Post-hoc (Tukey) test demonstrated significantly higher levels of H_2_O_2_ production by TACGauze (TGz) treated with citric acid and ascorbic acid or BIOGauze (BGz) versus either bleached cotton (B8-s2) or TGz alone. * *p* < 0.001 versus bleached cotton. ‡ *p* < 0.001 versus TGz.

**Figure 2:**
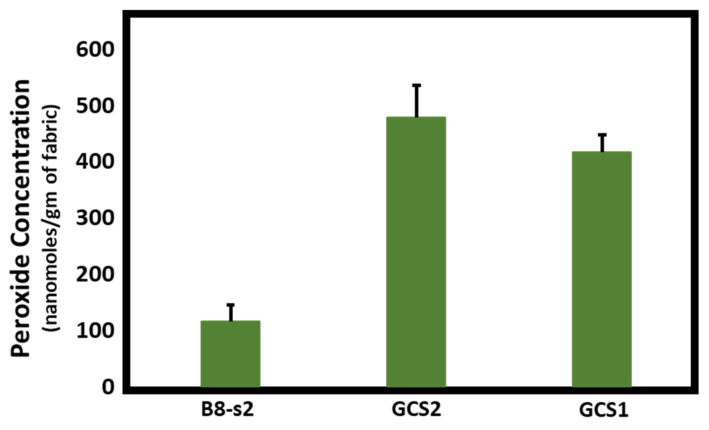
Effects of the addition of copper and ascorbic acid on hydrogen peroxide production. Fabrics (50 mg) were incubated overnight in 2 mL of H_2_O at room temperature. Aliquots were removed and H_2_O_2_ content was determined using the ferrous oxidation-xylenol orange assay in quadruplicate, *n* = 4. Error bars represent SEM. Results were analyzed by ANOVA. Post-hoc (Tukey) test demonstrated significantly higher levels of H_2_O_2_ production by 100% cotton woven fabric (bought commercially) treated with both copper and ascorbic acid (GCS2) (*p* < 0.001) or by cotton woven fabric treated with ascorbic acid only (GCS1) (*p* = 0.002) versus untreated bleached 100% cotton nonwoven (B8-s2) fabric.

**Figure 3:**
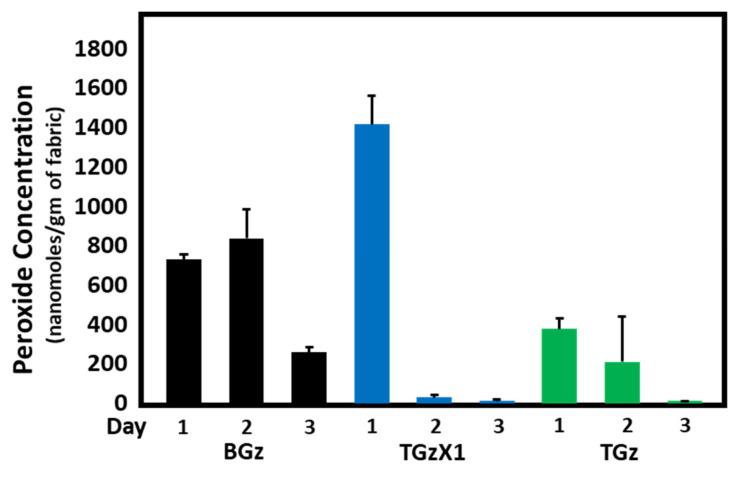
Comparison of hydrogen peroxide accumulation over a three-day period. Fabrics (50 mg) were incubated in H_2_O (2.5% weight/volume) at room temperature for 1, 2, and 3 days. Aliquots were removed and H_2_O_2_ content determined using the ferrous oxidation-xylenol assay, *n* = 4. Error bars represent SEM. ANOVA (Tukey) revealed that the levels of H_2_O_2_ generated by BIOGauze (BGz) were significantly higher on Day 2 compared to the those of the other two fabrics. Even though the peroxide levels generated with BGz declined significantly on Day 3, it was significantly higher than those of either the TGzX1, TACGauze acid-crosslinked treatment or the TGz, TACGauze untreated fabric, whose peroxide levels had fallen to essentially undetectable.

Figure 1, Figure 2 and Figure 3 demonstrate the hydrogen peroxide levels generated by the functional fabrics of this study. In this regard, it is interesting that the levels of hydrogen peroxide produced per gram of fabric are consistent with the antibacterial results shown in Table 2. As shown in Figure 1 and Figure 2, levels of hydrogen peroxide that correlate with the antibacterial activity were observed to be 250 to 550 nanomoles per gram of fabric. When assessed for the duration of hydrogen peroxide activity (Figure 3), the levels were also commensurate with the observed antibacterial activity present after three days in BGz. In formulation TGzX1, which consisted of ascorbic acid crosslinked to cellulose (Figure 3), the levels were robust at day one but fell off thereafter, most likely due to differences in the finishing conditions. 

### 2.3. Antiviral Activity

The treated cotton blend nonwoven BIOGauze (BGz) was tested for antiviral activity using TGz as a control to determine its efficacy against a challenge virus and one that has a high homology with the Severe Acute Respiratory Syndrome Coronavirus 2 (SARS-CoV-2) virus (88%). One of the routine challenge assays used to screen for any antiviral activity relating to textiles is the AATCC 100 test method (TM), “Assessment of Antibacterial Finishes on Textile Materials”, which is modified for viruses using the MS2 bacteriophage (MS2). MS2 is a non-enveloped positive-stranded RNA virus of the bacteriophage family *Leviviridae*. Its small size, icosahedral structure, and environmental resistance have made MS2 ideal for use as a surrogate virus in disinfectant studies. The permissive host cell for MS2 is *Escherichia coli*. The study showed that BGz reduced the viral load by 99.99 percent after one hour compared with the control and TGz, the BGz precursor, at time zero (Table 3). Notably, the human coronavirus, Strain 229E (HCoV-229E), was also used in the AATCC 100 TM modified for virus assay. The cell host, in this case, was MRC-5(ATCC CCL-171) from the human lung fibroblast cell line. We observed a 90% reduction for BGz and no reduction for TGz or the commercial N-95 mask sample at a time point of six hours (Table 4).

### 2.4. Ascorbic Acid Crosslinking to Cellulose in Cotton Fabrics

**Figure 4:**
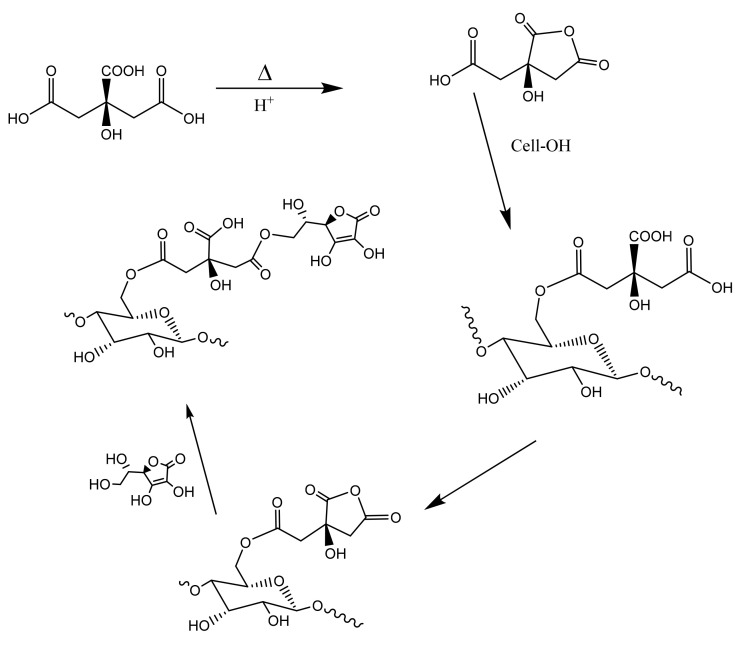
Schematic of the crosslinking reaction of ascorbic acid linked to cellulose. Ascorbic acid ((R)-5-[(S)-1,2,-dihydroxyethyl]-3,4-dihydroxyfuran-2(5H)-one) is reacted with citric acid under conventional pad-dry-cure conditions. The reaction is shown at the 1-hydroxy position of ascorbic acid to form the ester crosslinked analog of cellulose.

The formulation of ascorbic acid in cotton nonwovens gives rise to robust antimicrobial and antiviral levels of hydrogen peroxide. Thus, it is of interest to demonstrate that a covalently attached form of ascorbic acid and cellulose—e.g., an ascorbate–cellulose conjugate—elicits a comparable hydrogen peroxide activity. For this, we demonstrate here the crosslinking of ascorbic acid to cotton cellulose in similar fabrics to those described here. Figure 4 shows the chemistry of covalently attaching ascorbic acid to the cellulose in cotton by way of polycarboxylic acid. The polycarboxylic acid employed in this study was citric acid. It is important to note that polycarboxylic acid approaches to crosslinking cellulose in cotton have previously been extensively studied in the context of creating durable press fabric [9,10,11]. This work underscores the importance of acidic conditions or even the use of an acid catalyst such as sodium hypophosphite (SHP) [12]. As shown in Figure 4, the crosslinking proceeds through the acid-catalyzed formation of an acid anhydride.

**Figure 5:**
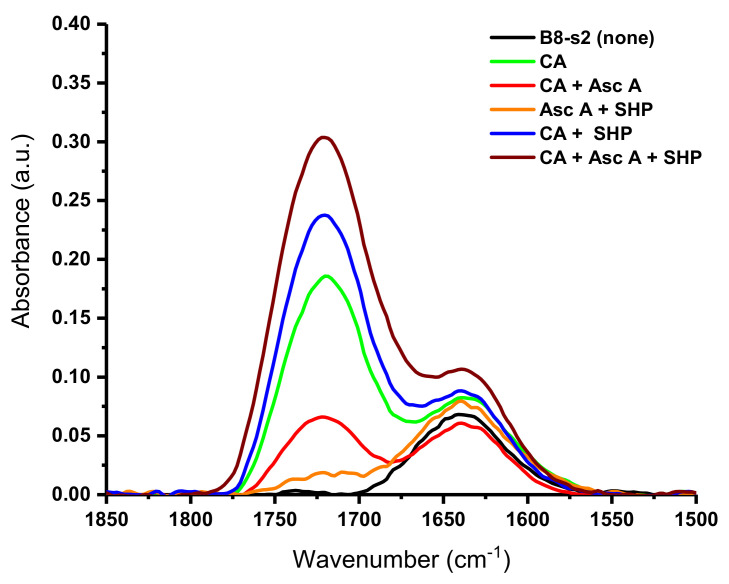
Fourier transform infrared spectra (1850–1500 cm^−1^) of crosslinked bleached 100% cotton nonwoven fabric (B8-s2); black line shows no treatment; others show treatment with 9% citric acid (CA), 5% ascorbic acid (Asc A), and 3% sodium hypophosphite (SHP), and their various control combinations.

To characterize the fabrics subjected to this treatment, we utilized the IR region displayed in Figure 5. It is notable that wide bands ranging between 1675 cm^−1^ and 1800 cm^−1^ appear for the fabrics treated in this study, while this same feature is missing for the untreated sample (Figure 5, black trace). This range, 1675 cm^−1^ to 1800 cm^−1^, coincides with the expected region of ester C=O groups [13]. For the fabric treated with citric acid, a moderately intense band appears with a peak point at 1721 cm^−1^. However, the treatment of a bleached nonwoven fabric with a combination of the citric acid and ascorbic acid produces a band at 1724 cm^−1^. Treatment with citric acid and the SHP catalyst increases the intensity of the band, and the peak point can be seen at 1721 cm^−1^. Moreover, the highest-intensity band in the ester region can be observed for the fabric treated with a combination of citric acid, ascorbic acid, and SHP, with a peak point at 1723 cm^−1^. This result is also correlated with the highest add-on. Fabrics treated with ascorbic acid or ascorbic acid with SHP display low-intensity ester bands at 1725 and 1723 cm^−1^, respectively. Remarkably, in this study the intensity of the ester bands follows the percent add-ons observed for the fabrics, with the ascorbic-acid-treated fabric showing the lowest-intensity band and add-on (1.56%) and the fabric treated with citric acid, ascorbic acid, and SHP showing the highest-intensity band and add-on (9.43%). Thus, given that thoroughly washed fabrics do not retain unreacted reagents, the correlation of IR bands to increased add-ons strongly suggests the formation of a covalent linkage between ascorbic acid and cellulose.

As described above, small differences in band peak positions can be observed for the various treatments. Fabrics treated with citric acid or the combination of citric acid and SHP have a band peak centered at 1721 cm^−1^. The similarity in these bands suggests that the addition of the SHP does not change the nature of the citrate cross-linkage but does increase its formation. In contrast, the addition of ascorbic acid to the citric acid and the SHP treatment mixture slightly shifts the position of the ester band to 1723 cm^−1^. The shifts in the band peak position to 1723 cm^−1^ for all fabrics treated with ascorbic acid further support the evidence for the covalent linkage of ascorbic acid. 

**Figure 6:**
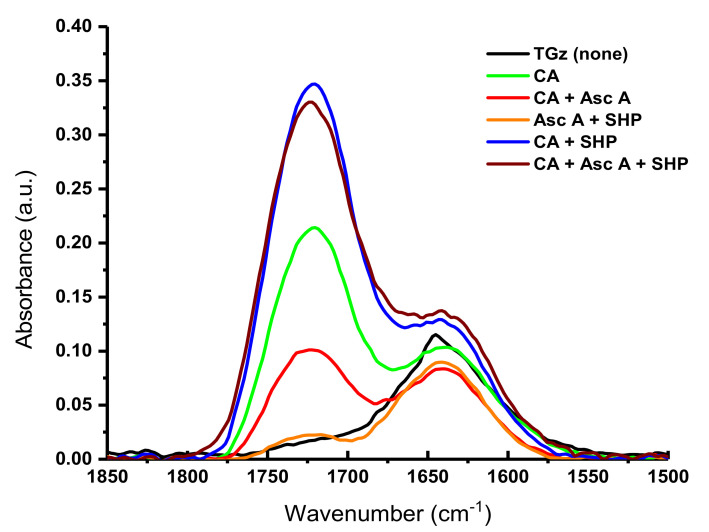
Fourier transform infrared spectra (1850–1500 cm^−1^) of crosslinked TACGauze (TGz); black line shows no treatment; other lines show treatment with 9% citric acid (CA), 5% ascorbic acid (Asc A), and 3% sodium hypophosphite (SHP) with their various control combinations.

Treated TGz gauze fabrics (Figure 6) show similar FTIR changes. A notable deviation for the TGz fabrics is the observed intensity of the ester bonds for the citric acid and SHP-treated fabric exceeding the intensity of the fabric treated with citric acid, ascorbic acid, and SHP. However, the close proximity of the add-on values for these two fabrics (6.82% for the former, 7.16% for the latter) likely accounts for this discrepancy.

**Figure 7:**
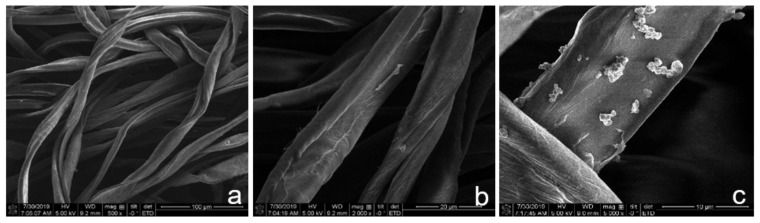
Scanning electron microscope(SEM) images of (**a**,**b**) TACGauze (TGz) untreated at magnifications of 500× and 1200×, respectively, and (**c**) BIOGauze (BGz) magnified 5000×.

Scanning electron micrographs of TGz and BGz are shown in Figure 7. The structures depicted in Figure 7 illustrate the interwoven disposition of the three fibers (bleached cotton, greige cotton, and polypropylene) of the TGz fabric resulting from hydroentanglement. The images of the deposited ascorbic acid crystals on greige cotton fibers in Figure 7c suggest that ascorbic acid is deposited as crystalline aggregates on the greige cotton fiber cuticle.

## 3. Discussion

### 3.1. Ascorbic Acid Mediated Hydrogen Peroxide Generation in Nonwovens and Their Antibacterial and Antiviral Activity

In this study, we delineate three types of finishing chemistries applied to cotton nonwovens to produce antimicrobial and antiviral fabrics. Pad/spray-drying and covalent cellulose crosslinking on spunlaced greige cotton nonwovens were found to produce an effective level of activity of up to 99.99% inhibition. The associated mechanism of action is thought to be the generation of hydrogen peroxide from the formulated fabrics. Thus, the antimicrobial activity of the ascorbic acid and cotton nonwoven formularies of this study is thought to be based on the classically characterized Fenton reaction. The molecular mechanism is well characterized: in the presence of metal ions such as copper or iron, ascorbic acid ((R)-5-[(S)-1,2,-dihydroxyethyl]-3,4-dihydroxyfuran-2(5H)-one) behaves as a pro-oxidant by cooperatively binding metal ions to form an organometallic bivalent complex, metal-dihydroxyfuranone complex (MDC); under aerobic conditions, MDC binds oxygen (O_2_), the core oxygen atoms of hydrogen peroxide, which can then dismutate by way of a protonated reactive oxygen species (ROS) to form hydrogen peroxide as the end product [14]. The initiation of this molecular mechanism in the spunlaced fabric is conceivable both in light of the levels of hydrogen peroxide demonstrated in the treated fabrics and consistent with the presence of the transition metal ions previously characterized in these types of cotton fabrics [7]. An interesting finding of this work is the relative efficacy of the ascorbic acid nonwoven formulations considering previously published studies that highlight their partial antibacterial efficacy. Moreover, the formulated fabrics function effectively in generating hydrogen peroxide levels commensurate with antimicrobial activity [15] for up to two days.

Our recent studies where we investigated the ability of cotton to generate hydrogen peroxide resulted in findings that are consistent with past reports on the generation of hydrogen peroxide in biological systems [16,17]: (1) cotton can generate hydrogen peroxide at levels that stimulate cell proliferation [18,19] and (2) the addition of ascorbic acid to greige cotton nonwoven fabrics results in robust levels of hydrogen peroxide associated with antibacterial activity [7]. Thus, this work demonstrates the efficacy of formulating unbleached cotton with low levels of add-ons (less than one percent) of ascorbic acid to produce antimicrobial efficacy against both Gram-positive and Gram-negative bacteria at a 99.99 percent inhibition of microbial growth [7]. It is also noteworthy that this antimicrobial design imparts a decidedly ‘green’ motif to the antimicrobial efficacy of the cotton fabric. The mechanism of antibacterial activity is thought to be the production of hydrogen peroxide.

### 3.2. Application to Wound Dressings

The development of robust hemostatic dressings that also have antimicrobial activity is desirable in pre-hospital medicine in both civilian and military scenarios [20]. This is especially the case for special forces operations that extend to remote and harsh parts of the world where the evacuation of casualties is measured in days rather than hours [21]. Microbial growth on textiles that come into contact with the body may double at a rate of 20–30 min, causing undesirable effects and creating the potential for the contamination of the user [22], especially when access to medical care is limited. In addition, an effective hemostatic dressing that also has robust antimicrobial activity is needed as seen with the evading virulent activity of *S. aureus*, which has evolved mechanisms to control blood coagulation. *S. aureus* is currently one of the deadliest infectious agents in the developed world, causing intravascular infections such as sepsis and infective endocarditis [23,24]. Thus, a robust, non-toxic antimicrobial that is effective for prolonged care is ideally suited for this purpose and should also (1) accelerates clot formation and halt the blood flow of hemorrhages in 2 min; (2) act as a barrier to microbial contamination and reduce bacterial colony formation; (3) remain in place for 72–96 h without tissue breakdown, reducing the need for frequent dressing changes; (4) conserve tissue viability by providing a moist environment; and (5) prevent premature wound closure and the formation of fistulae.

### 3.3. Application to Face Masks

Under controlled conditions, the formulation of nonwoven cotton with ascorbic acid results in antibacterial levels of hydrogen peroxide and is also commensurate with antiviral activity [7,8,25]. The mechanism is thought to be based on the process outlined above; in the presence of catalytic metals, ascorbate can have pro-oxidant effects, wherein the redox-active metal is reduced by ascorbate and then reacts with oxygen, producing superoxide. Superoxide dismutates to produce H_2_O_2_.

Thus, the mode of action and antiviral efficacy are relevant for face mask and barrier textile design. Moreover, numerous issues regarding the design and efficacy of face masks constructed with textiles are receiving increased attention [26,27]. It is apparent that the improvement of face mask efficacy will require highly controlled studies on a wide range of barrier fabrics to optimize the efficacy and safety assessments of new designs. For example, numerous studies have been performed on the survival of viral particles on different types of surfaces, both with and without disinfectants [28]. However, there are few reports in the literature on studies on the survival of active virus titer deposited from exhaled and inhaled breath, or a suitable surrogate that simulates respiration into the fabric medium. Thus, evaluating new textile designs that impart virucidal efficacy to cloth face masks in a safe, sustainable, and economical fashion has relevance for the current healthcare crisis brought on by SARS-CoV-2.

Considering the demonstration of the generation of hydrogen peroxide from cotton nonwovens in this study, the levels of hydrogen peroxide reported to be virucidal in the literature are within the range of levels we observed in the fabrics used in this study. The levels of hydrogen peroxide required to neutralize viral activity such as those found for SARS-CoV-2 have recently been reviewed [25]. Some reports have suggested that levels as low as 0.5% are adequate, while several reports indicate that levels of 1.5% are necessary. These levels are below those of bacteriostatic hydrogen peroxide. Levels of hydrogen peroxide of 3–4% are within the bacteriostatic range and are generally accepted to be virucidal. However, viruses would be expected to be less resistant to hydrogen peroxide. Hydrogen peroxide’s virucidal activity works through the oxidation of lipids and proteins to disrupt the viral replication cycle and prevent host cell entry. In this study, the hydrogen peroxide levels were well within this range. It is also important to note that the 90% inhibition observed for the coronavirus is expected to be the minimum level, since the results were blurred by host cell modifications that occurred during the virus assay—i.e., the lifting of E. coli cells in the Petri dish. Moreover, viruses generally do not have a protective mechanism against hydrogen peroxide as some bacteria do—e.g., catalase neutralization. It is also important to note that the cotton developed in these prototypes contains other constituents that have been reported to be antiviral—i.e., pectin and, to some extent, polyphenolic antioxidants.

## 4. Materials and Methods

### 4.1. Materials

The l-ascorbic acid and citric acid were purchased from Sigma Aldrich (now Millipore Sigma, Burlington, MA, USA). Sodium hypophosphite monohydrate (NaH_2_PO_2_ ● H_2_O) was from JT Baker. Ultrapure water (18 Ω), Millipore, was used as a solvent. The fabrics used were as follows: TACGauze(TGz) from H&H Medical; a blend of 50% greige cotton/30% bleached cotton/20% polypropylene; fine mesh gauze (FMGz); 100% bleached cotton (# 4-2915 inside roll 36″ 50yds roll from DeRoyal, Powell, TN, USA); 100% bleached cotton hydroentangled nonwoven fabric (B8-s2) produced at Southern Regional Research Center (SRRC) New Orleans, LA, USA; and 100% cotton plain weave printcloth fabric (GC), a quilting cotton fabric made by Dover Hill in “Burlap Pewter” purchased from Benartex.

### 4.2. Methods

#### 4.2.1. Treatment Application Methods

##### Pad-Dry and Pad-Dry Cure

The treatment of the fabrics: Fabric swatches were submersed and saturated in a solution volume of 20× the weight of the fabric. The saturated swatches were padded with a hand-cranked wringer (Calliger). Padding was repeated and the wet padded weight of the swatch was recorded. The swatches dried on a screen or metal frame in a force draft oven at 100–105 °C for 5–10 min or at120 °C for 3–5 min without tension. Swatches were not rinsed. The formulation swatches were equilibrated overnight before weight measurement.

For crosslinking ascorbic acid to cotton-blended fabrics, two process conditions were used which had differences in the reagent concentrations and cure temperatures and durations. Solutions 20× the weight of the fabric were made to treat the swatches along with the controls. Process condition I: 9% citric acid (CA) only; 5% ascorbic acid (Asc A) only; 9% CA + 5% Asc A; 9% CA + 3% sodium hypophosphite hydrate (SHP); 9% CA + 5% Asc A + 3% SHP; and 5% Asc A + 3% SHP. Swatches were saturated, padded, and dried for 3 min at 95 °C; then, they were cured for 5 min at 165 °C. Process condition II: 7% CA + 4.8% SHP only; 7% CA + 4.8% SHP + 1% Asc A. Swatches were saturated, padded, and dried for 3 min at 95 °C and cured for 3 min at 160 °C. All swatches were rinsed with deionized water. They were padded to remove excess water and dried in an oven at 100 °C for 3 min. They were weighed after equilibrating overnight.

##### Spray-Drying

Fabric swatches were attached vertically to a metal rack and sprayed until visible saturation using a generic spray bottle containing its corresponding solution: (1) 10 mM Asc A alone and (2) 10 mM Asc A with 2 mM copper(II)chloride (CuCl_2_). Excess solution was allowed to drip into a collecting pan until cessation. The swatches were placed on paper towels to dry flat overnight. They were weighed the following day after equilibrating overnight.

#### 4.2.2. Pilot Process Run: BIOGauze

The fabric formulation consisted of 0.95% (*w*/*w*) ascorbic acid and 0.06% (*w*/*w*) 1-hexanol added as a wetting agent. The process run treats the fabric using the following conditions: a fabric roll is padded with a wet pick up of 121%; it is processed for 20 s in an oven at a temperature (zone 1/zone 2) of 315 °F/320 °F (157 °C/160 °C), then an IR web temperature (zone 1/zone 2) of 240 °F/320 °F (115 °C/160 °C) at a line speed of 37 fpm. The targeted exit temperature was 320 °F/160 °C.

#### 4.2.3. Fourier-Transform Infrared (FTIR) Spectroscopy Analysis of Treated Fabrics

FTIR examinations of the TGz, B8-s2, and FMGz samples were performed using a Vertex 70 FT-IR spectrometer (Bruker Optics, Billerica, MA, USA) equipped with an attenuated total reflection (ATR) sampling accessory (Pike Technologies, Madison, WI, USA) and a diamond-ZnSe reflective crystal. Samples were placed on top of the ATR crystal and secured with a metal clamp. Each sample was examined at 5 different points chosen at random. A total of 32 scans were measured between 3800 and 600 cm^−1^, with a resolution of 4 cm^−1^ for each replicate. Spectra are presented as the average values for the five replicates. Additionally, the spectra obtained for each sample were processed with the OPUS spectroscopy software (version 6.5) (Bruker Optics, Billerica, MA, USA) using concave rubber-band correction with the option to exclude CO_2_ bands selected for baseline correction and normalized with the min–max normalization method (OPUS). No ATR correction or atmospheric compensation was performed.

#### 4.2.4. Antimicrobial Activity

Selected fabrics were submitted to Situbiosciences (Wheeling, IL, USA) for fabric testing using the AATCC100 test method for textiles. AATCC TM 100 test method was designed to measure the antimicrobial properties of textiles or absorbent materials incubated with selected microorganisms. In this method, the microorganism inoculum is incubated in contact with the test sample for a duration of 24 h without drying. Following this exposure, the inoculated microorganisms are recovered and the concentration of the organisms is determined. The antimicrobial performance is determined by the comparison of the recovered organisms from the test samples at time 0 and the treated material after selected time points—in this case, 24 h—and is reported as a percent value relative to the control sample material.

#### 4.2.5. Antiviral Testing

Selected fabrics were submitted to Microchem Laboratory (Round Rock, TX, USA) for antiviral fabric testing with the AATCC-100 test method for antibacterial finishes on textile materials modified for viruses. The screening assay used Bacteriophage (MS2) ATCC15597-B1 with *Escherichia coli* 15597 as the permissive host cell system. The assay was repeated with two controls and the test microorganism was human coronavirus, strain 229E, ATCC VR-740, with the host cell MRC-5 (ATCC CCL-171) human lung fibroblast cell line. The procedures used at Microchem Laboratory are summarized below.

##### AATCC-100 Test Method Modified for Viruses Using MS2 Bacteriophage

Test samples/articles were cut into 4.8 cm-diameter circles (5/stack). The control fabric was steam-sterilized. The inoculum was prepared from a frozen stock culture by diluting it to the specified concentration using phosphate-buffered saline. Once prepared, the inoculum was plated to confirm the starting concentration. A milliliter of inoculum was added to each stack of fabric articles. A subset of control articles was harvested following inoculation to determine the starting concentration on the fabric. Following inoculation, the test articles and parallel control articles were incubated in closed sealed Petri dishes inside a sealed plastic bag/container under incubation conditions optimal for the microorganism, typically 36 °C. Upon the completion of the contact time(s), which was dictated by the length of incubation, the test and control articles were harvested. Carriers were harvested by aseptically folding stacks and placing them into neutralizer media, D/E Broth, then mixing them for ~1–2 min by vortex. The neutralizer was then plated using standard dilution and plating techniques. Enumeration plates were incubated at the appropriate conditions for 12–24 h. Sterility controls, including the test and control articles, were used on each day of testing. The microorganisms used in the study were checked for purity.

##### AATCC-100 Test Method Modified for Viruses Using Human Coronavirus

The stock virus was thawed and was not supplemented with an organic soil load. From the test samples, 1-inch squares were cut and placed into sterile plastic Petri dishes. To begin contact time, a 0.100–0.200 mL volume of virus was inoculated onto each sample surface while ensuring the equal distribution of the swatches. The Petri dish was then covered for the duration of the contact time. At the completion of the contact time, each sample was aseptically transferred to a sterile conical tube containing an appropriate volume of neutralization media and vortexed. After vortexing, a 0.1 mL aliquot was used in a series of 10-fold dilutions of appropriate test medium. Each dilution was inoculated into the appropriate host cells in quadruplicate. The log and percent reduction in the viral titer were calculated using the test and recovery control titer results. The control swatch was tested in the same manner as the test sample swatches. For the cytotoxicity control, the test procedure was followed with the exception that the aliquot of the test medium was used to inoculate the control in lieu of the virus. To confirm test substance neutralization, an aliquot of the dilutions prepared in the cytotoxicity control was inoculated into the host cells. The cells were incubated at the appropriate test conditions for approximately 7 days and microscopically observed the test virus for cytotoxic effect and the virus titer.

#### 4.2.6. Determination of Hydrogen Peroxide Production: FOX 1 Assay

Hydrogen peroxide produced by the treated samples was determined using the ferrous oxidation with xylenol orange (FOX1) assay method 1 [29,30]. Briefly, stock solutions were made from the reagents to create the substrate solution to add to the sample aliquot in order to measure peroxide production spectrophotometrically. Approximately 50 mg of the sample swatch was incubated overnight at room temperature in 2.0 mL of deionized water. Aliquots of 50 µL in quadruplicate of the resulting sample swatch solution were used for the assay. The substrate solution consisted of final concentrations of 25 mM sulfuric acid (H_2_SO_4_), 100 µM xylenol orange, 100 mM sorbitol, and 250 µM ferrous ammonium sulphate in water. The sample aliquot, 50 µL, was added to 950 uL of substrate solution and shaken. The absorbance was measured at 560 nm at one-minute intervals for six minutes. A standard curve of hydrogen peroxide (H_2_O_2_) for 0–10 µM concentrations was used to calculate the unknown peroxide content in the test samples.

#### 4.2.7. Scanning Electron Microscopy (SEM)

The imaging of the nonwoven fabrics’ morphology and fine structure was conducted using a JEOL JSM-6610 LV SEM scanning electron microscope at the Shared Instrumentation Facility at Louisiana State University. Small fabric swatches were mounted on stubs, coated with gold/palladium, and image scanned under high vacuum with an accelerated voltage of 5 kV and a magnification ranging from 500 to 5000×.

## 5. Conclusions

To date, there have been no reports on the use of ascorbic acid as an antibacterial in commercially produced textiles. Despite the low cost, health-promoting effects, and non-toxic nature of ascorbic acid, it has so far not been used in commercial textile applications as an antimicrobial. Accordingly, we demonstrate that the application of ascorbic acid to unbleached cotton textiles results in the complete inhibition of both Gram-negative and Gram-positive bacteria activity commensurate with levels necessary for commercial use.

In examining these textiles, we explored a variety of potential mechanisms for generating hydrogen peroxide in cotton fibers. Moreover, we found that H_2_O_2_ production by greige cottons can be further modulated by the simple addition of ascorbate. It is also notable that ascorbate equalizes the hydrogen peroxide activities of cotton in hydroentangled, needle-punched, and field cotton samples within the concentration range reported for cell signaling [18].

Moreover, the use of vitamin C under Fenton catalysis conditions provides a system that could be used for extended hydrogen peroxide generation in greige cotton nonwovens [7,18].

## Figures and Tables

**Table 1:** Description of fabrics.

Fabric Name and Description	Fabric Treatment
TACGauze (TGz)	None
BIOGauze (BGz)	Pilot-scale pad-dry ascorbic acid on TACGauze
TACGauze, crosslinked (TGzX1)	Pad-dry cure of citric and ascorbic acid (crosslinking)
TACGauze, sprayed (TGzS1)	Spray-dried 10 mM ascorbic acid
TACGauze, sprayed (TGzS2)	Spray-dried 10:1 mM ascorbic acid and copper(II) chloride (CuCl_2_)
TACGauze, lab (TGzL)	Pad-dry of 10 mM ascorbic acid (lab scale)
TACGauze, lab-pilot (TGzPL)	Pad-dry of 10 mM ascorbic acid (lab-pilot scale using padding strength of 5-30psi)
Plain weave woven 100% cotton fabric, sprayed (GCS1)	Spray-dried 10 mM ascorbic acid
Plain weave woven 100% cotton fabric, sprayed (GCS2)	Spray-dried with 10:1 mM ascorbic acid and copper(II) chloride (CuCl_2_)
100% bleached cotton nonwoven (B8-s2)	None
100% cotton fine mesh gauze crosslinked (FMGzX1)	Bleached 100% cotton gauze pad-dry-cured with citric and ascorbic acid (crosslinking)

**Table 2:** Antimicrobial results of prototypes, pilot, and lab-treated samples using the AATCC 100 test method ^a^ against two standard microorganisms.

Sample Description	*K. Pneumonia* (4352)	*S. aureus* (6538)
	% Reduction after 24 h	
(TGz)	0	0
(BGz)	99.99	99.99
(TGzS1)	99.9	99.9
(TGzS2)	99.9	99.9
(TGzL)	99.99	99.99
(TGzPL) 5psi	99.99	99.99
(TGzPL) 30 psi	99.35	99.99
(GCS1)	0	0
(GCS2)	0	94.8
(FMGzX1)	99.99	99.99

^a^ performed by Situbiosciences.

**Table 3:** Preliminary antiviral screening ^a^ result of prototypes tested against surrogate virus, MS2 bacteriophage.

Carrier	Contact Time	Average PFU/Carrier ^b^	% Reduction	Log_10_ Reduction
Control	5 min	1.78 × 10^7^	N/A	N/A
(Time zero)	1 h	1.47 × 10^6^		
	3 h	1.47 × 10^6^		
Control	5 min	2.25 × 10^7^	N/A	N/A
	1 h	1.50 × 10^6^		
	3 h	2.10 × 10^6^		
TACGauze	5 min	3.63 × 10^6^	79.55	0.69
	1 h	9.67 × 10^5^	34.09	0.18
	3 h	1.83 × 10^6^	No Reduction	No Reduction
BIOGauze	5 min	7.00 × 10^6^	60.60	0.40
	1 h	2.13 × 10^2^	99.99	3.84
	3 h	1.47 × 10^6^	>99.99932	>5.17

^a^ AATCC100 test method for antibacterial finishes on textile materials modified for viruses was used to test for antiviral activity using the surrogate virus, MS2 bacteriophage ATCC 15597-B1 (performed by Microchem Laboratory). ^b^ The limit of detection for this assay was 1.00 × 10^1^ PFU/carrier; results are reported as <1.00 × 10^1^ PFU/carrier in the table above.

**Table 4:** Preliminary results of the virucidal activity of prototypes tested against human coronavirus ^a^ microorganism with a contact time of 6 h.

	TCID_50_ per 0.1 mL ^b^	% Reduction	Log Reduction
Virus titer	5.50 log_10_	-	-
Virus (time zero)	5.50 log_10_	-	-
Virus control	3.50 log_10_	-	-
TACGauze	4.50 log_10_	No reduction	No reduction
BIOGauze	2.50 log_10_	90%	1.00
N95 mask material	5.00 log_10_	No reduction	No reduction

^a^ AATCC100 test method for antibacterial finishes on textile materials modified for viruses was used to test for antiviral activity using human coronavirus, strain 229E, ATCC VR-740; host cell MRC-5 (ATCC CCL-171) (performed by Microchem Laboratory). ^b^ TCID_50_ (Tissue Culture Infectivity Dose) represents the endpoint dilution where 50% of the host cell monolayers exhibit cytotoxicity, which was determined using the Spearman–Kärber method.
